# Draft Genome Sequence of Low-Passage Clinical Isolate *Porphyromonas gingivalis* MP4-504

**DOI:** 10.1128/genomeA.00256-16

**Published:** 2016-04-07

**Authors:** Thao T. To, Quanhui Liu, Michael Watling, Roger E. Bumgarner, Richard P. Darveau, Jeffrey S. McLean

**Affiliations:** a Department of Periodontics, University of Washington School of Dentistry, Seattle, Washington, USA; b Department of Microbiology, University of Washington, Seattle, Washington, USA

## Abstract

We present the draft genome of *Porphyromonas gingivalis* MP4-504, a low-passage clinical isolate obtained from a periodontitis patient. The genome is composed of 92 contigs for a length of 2,373,453 bp and a G+C of 48.3%. The *traA-Q* conjugative transfer locus is genetically distinct from W83 but highly similar to ATCC 33277.

## GENOME ANNOUNCEMENT

The Gram-negative oral anaerobe *Porphyromonas gingivalis* is highly associated with periodontal disease and inflammation (1, 2). Considered a “keystone pathogen,” *P. gingivalis* contributes to development and stabilization of a dysbiotic microbial community, leading to chronic inflammatory responses indicative of periodontitis (3–5). However, the various genetic mechanisms of this species are not fully understood and increased availability of genomic sequences is needed. Here, we report the draft genome sequence of low-passage clinical isolate MP4-504, which properties include stable adherence to oral streptococci (6), enhanced invasion of gingival epithelial cells (GECs) (7), strong inhibition of IL-8 production by GECs (8), and the ability to transfer DNA by conjugation at high efficiencies (9).

MP4-504 was originally sampled from the University of Washington Graduate Periodontics Clinic from the periodontal pocket (8-mm probing depth) of a chronic periodontitis patient and immediately transported to anaerobic conditions. The sample was then serially diluted on blood agar plates for bacterial isolation and anaerobically incubated at 35°C for 7 days before preliminary biochemical identification (10) and storage in a −80°C freezer collection.

For this study, isolate MP4-504 was grown as previously described and subjected to two additional passages beyond the primary freezer stock (7). Genomic DNA was extracted using the Qiagen DNeasy blood and tissue kit. Paired-end 300 bp reads were sequenced using the Illumina MiSeq platform. All quality-trimmed reads were *de novo* assembled using SPAdes v3.61 using default parameters (11, 12).

The final assembly consists of 92 contigs with a length of 2,373,453 bp (*N_50_*, 57,689 bp) and overall G+C content of 48.3%. Gene annotation using the Prokaryotic Genome Automatic Annotation Pipeline (PGAAP) provided by the National Center for Biotechnology Information (NCBI) identified a total of 2,070 genes, consisting of 1,891 coding sequences, 47 tRNAs, 3 rRNAs, and 3 clustered regularly interspaced short palindromic repeats (CRISPRs). MP4-504 shares 98.84% average nucleotide identity (ANI) (13) with closest phylogenetic neighbor, W83 (14), and 98.67% ANI with type strain ATCC 33277 (15). MP4-504 also shares 98.62% ANI with JCVI SC001, a *P. gingivalis* isolate recovered from a hospital sink biofilm (16).

MP4-504 is also capable of transferring shuttle vectors of *Bacteroides-Escherichia coli* origin to *E. coli* by conjugation, similar to ATCC 33277 and unlike type strain W83 (9, 17). Consistent with these findings, comparative analysis of the MP4-504 draft genome using the Rapid Annotation and Subsystem Technology (RAST) (18) server against the genomes of other *P. gingivalis* strains revealed that MP4-504 bears a similar genomic region to cTnPg1 reported in ATCC 33277 (17), with similarity of 93.3% at the protein level. When compared to W83, this MP4-504 *traA-Q* region bears only 42.8% similarity. Likewise, this region contains a gene encoding conjugative transposon protein, TraP, necessary for the conjugation of plasmids in ATCC 33277 but missing in W83. Further comparative analysis with other available *P. gingivalis* genomes will expand our understanding of genetic mechanisms underlying this bacterium’s pathogenesis.

### Nucleotide sequence accession numbers.

This whole-genome shotgun project has been deposited at DDBJ/EMBL/GenBank under the accession no. LOEL00000000. The version described in this paper is version LOEL01000000.
